# modPDZpep: a web resource for structure based analysis of human PDZ-mediated interaction networks

**DOI:** 10.1186/s13062-016-0151-4

**Published:** 2016-09-21

**Authors:** Neetu Sain, Debasisa Mohanty

**Affiliations:** Bioinformatics Center, National Institute of Immunology, Aruna Asaf Ali Marg, New Delhi, 110067 India

**Keywords:** PDZ domain, C-terminal peptides, Structure based approach, Protein-protein interaction networks

## Abstract

**Background:**

PDZ domains recognize short sequence stretches usually present in C-terminal of their interaction partners. Because of the involvement of PDZ domains in many important biological processes, several attempts have been made for developing bioinformatics tools for genome-wide identification of PDZ interaction networks. Currently available tools for prediction of interaction partners of PDZ domains utilize machine learning approach. Since, they have been trained using experimental substrate specificity data for specific PDZ families, their applicability is limited to PDZ families closely related to the training set. These tools also do not allow analysis of PDZ-peptide interaction interfaces.

**Results:**

We have used a structure based approach to develop modPDZpep, a program to predict the interaction partners of human PDZ domains and analyze structural details of PDZ interaction interfaces. modPDZpep predicts interaction partners by using structural models of PDZ-peptide complexes and evaluating binding energy scores using residue based statistical pair potentials. Since, it does not require training using experimental data on peptide binding affinity, it can predict substrates for diverse PDZ families. Because of the use of simple scoring function for binding energy, it is also fast enough for genome scale structure based analysis of PDZ interaction networks. Benchmarking using artificial as well as real negative datasets indicates good predictive power with ROC-AUC values in the range of 0.7 to 0.9 for a large number of human PDZ domains. Another novel feature of modPDZpep is its ability to map novel PDZ mediated interactions in human protein-protein interaction networks, either by utilizing available experimental phage display data or by structure based predictions.

**Conclusions:**

In summary, we have developed modPDZpep, a web-server for structure based analysis of human PDZ domains. It is freely available at http://www.nii.ac.in/modPDZpep.html or http://202.54.226.235/modPDZpep.html.

**Reviewers:**

This article was reviewed by Michael Gromiha and Zoltán Gáspári.

**Electronic supplementary material:**

The online version of this article (doi:10.1186/s13062-016-0151-4) contains supplementary material, which is available to authorized users.

## Background

PDZ domains are peptide recognition modules (PRMs) which recognize short 5-6 residue peptides present in C-terminal of their interaction partners. They are known to be involved in many important biological processes and disruption of PDZ domain mediated interactions leads to several diseases like cancer and neurological disorders [1]. Despite of having low affinity, they exhibit high degree of specificity. Earlier they have been classified into three classes based on their binding specificity: class1 (XϕXϕ), class2 (X[T/S]Xϕ) and class3 (X[E/D]Xϕ), where ϕ represents any hydrophobic amino acid [2, 3]. Their specificity landscape has been further explored by the phage display experiments using random peptide libraries and these studies have defined 16 specificity classes of PDZ domains [4]. In view of the small interaction interface mediated by peptides, they are targets for intervention by small molecules or peptidomimetic modulators [5]. Even though available experimental data on peptide binding specificity of PDZ domains have provided valuable clues for deciphering their complex specificity landscape, for prediction of genome wide interaction partners of PDZ domains, it is necessary to develop fast computational approaches which can quickly scan large number of potential binding partners and will also permit analysis of structural details of binding interfaces.

Among the computational tools currently available for prediction of PDZ binding partners, PDZpepInt [6] uses only sequence information of the substrate peptide and it has been trained using experimental data on binding specificity for 94 PDZ domains in total from human, mouse, worm and fly obtained from PDZBase [7], 54 human PDZ domains from phage display data and also additional data on mouse PDZ domains from microarray studies [4, 8]. Based on clustering of sequence of the query PDZ domain along with sequences of PDZ domains used in training, PDZpepInt permits peptide binding prediction for other human PDZ domains as well. However, out of 264 PDZ domains present in human genome, PDZpepInt can predict substrates for only 105 human PDZ domains. In contrast to the sequence based approach used in PDZpepInt, the POW server developed by Hui et al [9] uses a structure based approach, but it also utilizes machine learning and it has been trained using experimental data on PDZ binding specificity of 25 human and 58 mouse PDZ domains [4, 8]. Even though POW server of Hui *et al* can in principle predict substrates for about 218 human PDZ domains, it has not been benchmarked on newly available experimental binding specificity data [10]. Secondly POW server does not provide any tool for structural analysis of binding interface, even if it uses a structure based approach for prediction of PDZ substrates. In a recent work from our group, we demonstrated that structure based approach in combination with residue based statistical pair potentials is a practical approach for genome wide scan of PDZ interaction partners and prediction accuracy of this approach was benchmarked using high throughput proteome array data on 79 mouse PDZ domains and 217 mouse proteome derived C-terminus peptides [8, 11]. In this work, we have developed modPDZpep web server to predict the interaction partners of human PDZ domains using a structure based approach and attempted to benchmark prediction accuracy of this structure based approach using phage display and other available experimental data for human PDZ domains. Unlike PDZpepInt and POW, modPDZpep does not require experimental binding specificity data for training. Hence, all the available binding specificity data has been used as test set for validating the structure based prediction approach used in modPDZpep. modPDZpep provides user friendly graphical user interfaces for structural analysis of modeled PDZ peptide complexes. Secondly, since modPDZpep uses statistical pair potentials for scoring of binding energy, it is fast enough for genome scale analysis of PDZ interaction networks. The backend databases of modPDZpep have also stored experimental specificity data on various human PDZ domains obtained from phage display studies. Using phage display data and structure based prediction modPDZpep can also identify novel PDZ mediated interactions in human protein-protein interaction (PPI) network, many of which are not present in databases like STRING [12].

## Results and discussion

### modPDZpep uses a homology modeling approach in combination with statistical pair potentials

**Fig. 1:**
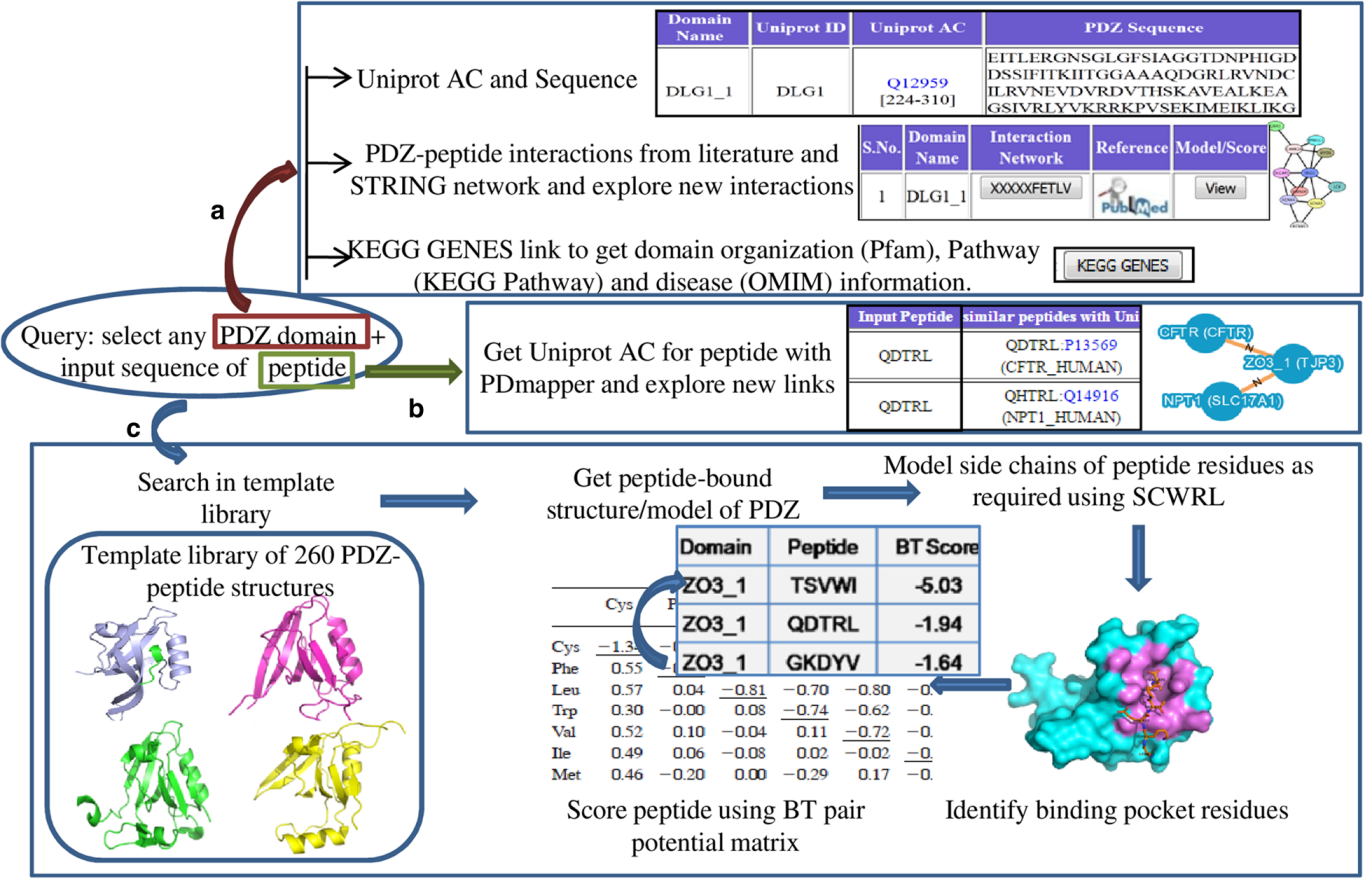
Overview of different features of modPDZpep. **a** modPDZpep has cataloged information on sequence, 3D structure and experimental substrate specificity data for all human PDZ domains. It also provides links to STRING database and KEGG GENES for analyzing functional interaction network. **b** PDmapper module aids in mapping of PDZ binding phage display peptides to UniProt AC and exploring new interactions involving PDZ domains. **c** The peptide bound structure of any human PDZ domain with any query peptide can be modeled and binding energy can be computed with modPDZpep

As depicted in Fig. 1, our structure based approach essentially follows a homology modeling protocol. It requires peptide bound structures of PDZ domains as template for modeling the query peptide in complex with a selected PDZ domain. The conformation of the main chain of substrate peptide is retained same as template and then side-chains are generated using SCWRL rotamer library [13] as per the sequence of the query peptide. Our approach is based on the assumption that backbone structural variations both in the PDZ domains and the bound peptides are minimal. Analysis of crystal structures of PDZ domains and PDZ-peptide complexes in earlier studies from our group as well as others [11, 14] have revealed that, backbone RMSDs of PDZ domains show good correlation with sequence similarities. It has also been shown that backbones of bound peptides superpose well when corresponding PDZ domains are superimposed. Therefore, in coarse grained modeling of PDZ peptide complexes for quick screening of binding partners assumption about minimal structural variations is justified. However, this does not imply that conformational flexibility of PDZ-peptide complexes have no role in peptide recognition.

After modeling the structure of the desired PDZ-peptide complex, the binding energy of the peptide in the PDZ pocket is scored using Betancourt-Thirumalai (BT) residue based statistical pair potential matrix [15]. BT (Betancourt-Thirumalai) pair potential is a knowledge based scoring function derived from observed contact frequencies between various amino acid residues and is expressed as 20x20 contact potential matrix corresponding to all possible amino acid pairs. The binding energy for each PDZ-peptide complex is scored as sum of the interactions between all residue pairs between the peptide and PDZ domain which are at a distance less than 4.5 Å. Residue based statistical pair potentials are less compute intensive and allow the fast screening of potential peptide substrates of PDZ domains and are thus suitable for genome scale analysis of interaction network. A number of different residue based statistical pair potentials are available in the literature and they have been successfully used in several protein structure prediction studies [16, 17]. They all have been derived from observed contact frequency of different pairs of amino acids in a non-redundant set of crystal structures in PDB. Basic assumption in derivation of all these pair potentials is that the observed contact frequency of an amino acid pair when normalized with respect to the expected frequency in a suitable reference state would present interaction energy between the residue pair. The major difference in various pair potentials is in the choice of reference state. Miyazawa-Jernigan (MJ) potential [18] was derived using solvent as reference state while Betancourt-Thirumalai (BT) pair potential used a solvent like molecule Thr as reference state. Therefore, it has been suggested that while MJ matrix gives more weightage to hydrophobic interactions, BT matrix can also represent hydrophilic interactions more accurately [19]. In our earlier study BT matrix was found superior to MJ matrix in identification of interaction partners of MHCs, kinases and mouse PDZ domains [11, 20–22]. Therefore, we preferred to use BT pair potential [15] for scoring PDZ-peptide complexes in modPDZpep.

### Structures of almost all human PDZ domains can be modeled

**Fig. 2:**
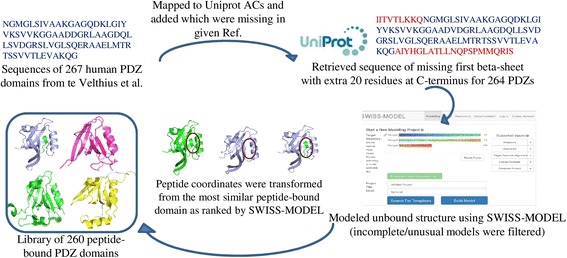
Generation of template library containing peptide bound PDZ domains. The set of 267 human PDZ domains identified by te Velthius et al. [24] were mapped onto proteins in UniProt. UniProt ACs could be obtained for 264 PDZ domain containing proteins. For each of these 264 PDZ domains a stretch of residues N-terminus to the domain sequences identified by te Velthius et al. [24] were also included as that region harbored a structurally conserved β sheet. The structural models for each of these 264 PDZ domains in absence of the bound peptide were obtained from SWISS-MODEL. Each of these 264 ligand free PDZ structural models were aligned with the available 38 peptide bound crystal structures of PDZ domains and from nearest peptide bound PDZ domain, the peptide coordinates were transformed after optimal superposition of apo and holo PDZ domain structures. Using this protocol peptide-bound structural model could be obtained for 260 human PDZ domains and remaining four were excluded as they lacked complete 3D structure of the PDZ domain

**Fig. 3:**
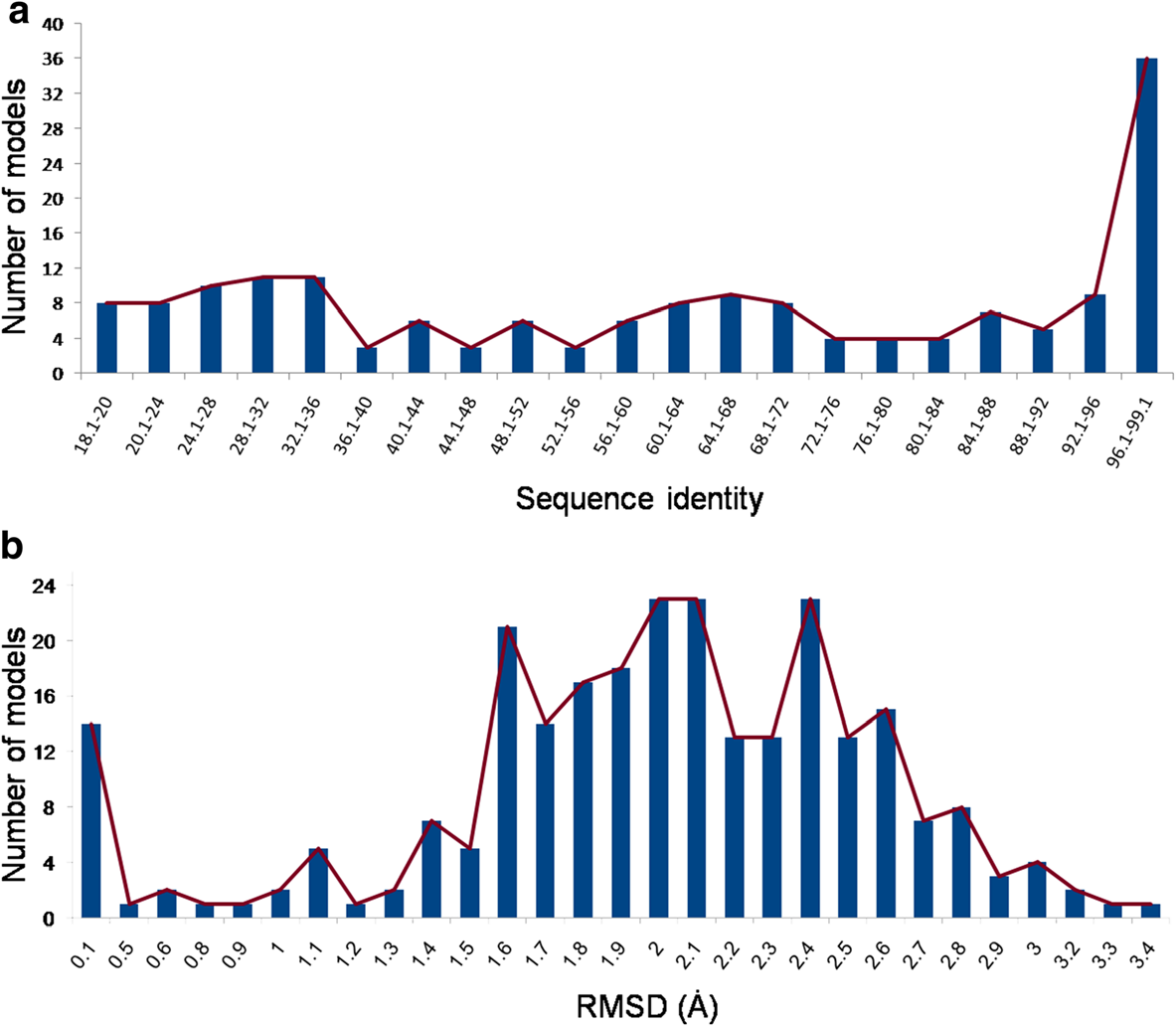
Histograms showing quality of structural models obtained for human PDZ domains. **a** Number of models is plotted against the sequence identity between the target and template PDZ sequences. 118 out of 169 PDZ domains have >40 % sequence identity to the PDZ domain structures used as template for generating the structural models, while crystal structures were available for 91 human PDZ domains. **b** Number of models is plotted against the RMSD of PDZ Structural models to the peptide bound templates calculated with TM-align. Most of the PDZ domains show <2.5 Ȧ RMSD to the peptide bound templates

For 16 human PDZ domains crystal structures were available for PDZ-peptide complexes and hence for these 16 PDZ domains bound peptide of any given sequence can be modeled by *in silico* mutation of side chains of bound peptides. For 75 PDZ domains crystal structures were available for PDZ domain alone, hence peptide bound structures were modeled by superposition of the PDZ domain on most similar PDZ-peptide crystal structure and by transforming the coordinates of the bound peptide from crystal structure. For the remaining 169 PDZ domains for which no crystal structures were available, homology models were built using SWISS-MODEL [23] and then peptide bound PDZ models were obtained by coordinate transformation from most similar crystal structures of PDZ-peptide complexes. This led to generation of template library of 260 PDZ-peptide complexes out of 264 human PDZ domains obtained after mapping the PDZ sequences retrieved from te Velthius et al. [24] to Uniprot accession numbers [25]. These 260 PDZ-peptide complexes can be used to model any peptide sequence in binding pocket of a given PDZ, but the length of the bound peptide will be restricted to the length of peptide present in the template PDZ-peptide crystal structure (Figs. 2 and 3). However, since recognition motif for most PDZ domains being around 5 residues, peptide length will not be a constraint in estimating binding energy of any peptide with these 260 human PDZ domains. Thus our template library covers ~98 % of the human PDZome and interactions for them can be predicted using structure based approach. The human PDZ-mediated interaction network can be structurally annotated using this template library.

### Benchmarking of modPDZpep

**Fig. 4:**
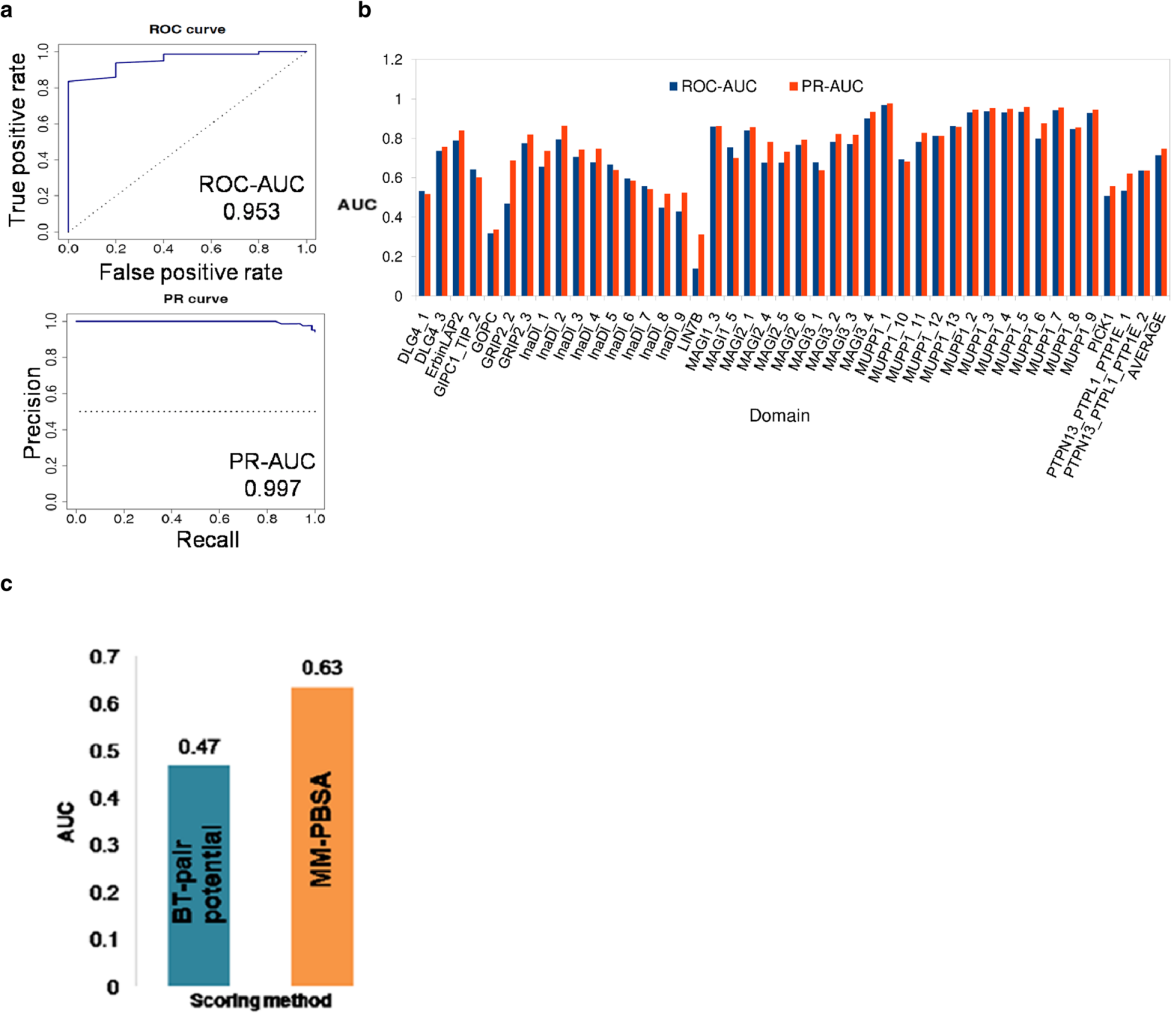
**a** ROC-curve for predictions of binder and non-binder peptides for MUPP1_1 PDZ domain is shown in upper panel with AUC (area under curve) value of 0.953 and lower panel shows PR-curve for same domain with AUC value of 0.997. **b** Performance of modPDZpep for prediction of binder and non-binder peptides for 43 human PDZ domains estimated using AUC values of ROC and PR curves. ROC and PR-AUC values for all 43 PDZ domains are present in Table 1. **c** Typical example (GRIP2_2 PDZ) of improvement in performance upon using atomistically detailed MM-PBSA approach for scoring of binding energy

modPDZpep has cataloged experimentally characterized interaction data on 199 human PDZ domains with 2898 peptides by compiling data from various high throughput studies reported in literature [4, 10, 26, 27]. Since our method is not training-based, experimental data can be solely used for testing the prediction accuracy of modPDZpep. Therefore, we wanted to investigate the predictive power of the statistical pair potential based structural modeling approach for human PDZ domains using this experimental specificity data. Out of these interactions, a subset of PDZ domains for which both positive and real negative interaction data were available was used for benchmarking (see Methods). Receiver Operating characteristic (ROC) and precision-recall (PR) analysis were used to assess the performance of our approach. Since real negative dataset for PDZ domains will be smaller in size compared to positive dataset of known binders, one often deals with highly imbalanced data set and several strategies have been proposed for ROC analysis of imbalanced dataset [28]. We carried out re-sampling of the data by randomly selecting equal number of positive and negative peptides in each set and computing separate area under curve (AUC) values for each set, and finally average AUCs was recorded for each PDZ domain. Figure 4a shows typical ROC curve and Precision-Recall (PR) plot for the predictions on MUPP1_1 PDZ domain by modPDZpep and they have AUC values of 0.953 and 0.997 for ROC and PR respectively, indicating high prediction accuracy. The AUC values of the ROC and PR curves for the predictions on all the 43 different PDZ domains by modPDZpep are shown in Fig. 4b and AUC values for those plots are given in Table 1. In cases where number of non-binders was much less compared to the number of binders, AUC was calculated by dividing the binders into multiple sets such that, approximately equal number of binder and non-binder were in each set and average AUC values are reported. The obtained average values of ROC-AUC of ~0.71 and PR-AUC of ~0.75 for this set of 43 PDZ domains indicate that it can successfully predict the human PDZ-peptide interactions. We also calculated other statistical parameters like sensitivity/specificity, accuracy, false positive rate (FPR), positive prediction value (PPV) and F1 score etc (Table 2) at a score cut off of -2.11 by randomly selecting equal number of binder and non-binder peptides (~290) for 34 domains having ROC-AUC >0.6. For this balanced set of binder and non-binder peptides from 34 PDZ domains, the average ROC-AUC was 0.79 and PR-AUC was 0.8, thus indicating a good representative data set. It was encouraging to note that, modPDZpep had sensitivity, specificity, accuracy and PPV values close to 70 %.

**Table 1:** ROC-AUC and PR-AUC values for 43 Human PDZ domains with real negative interaction data

Domain	ROC-AUC	PR-AUC	#Binder	#Non-binder
DLG4_1	0.53	0.52	71	5
DLG4_3	0.74	0.76	89	5
ErbinLAP2	0.79	0.84	43	13
GIPC1_TIP_2	0.64	0.60	26	13
GOPC	0.32	0.34	13	8
GRIP2_2	0.47	0.69	6	5
GRIP2_3	0.78	0.82	8	5
InaDl_1	0.66	0.74	48	7
InaDl_2	0.80	0.86	53	7
InaDl_3	0.71	0.74	67	7
InaDl_4	0.68	0.75	40	7
InaDl_5	0.67	0.64	43	7
InaDl_6	0.60	0.58	41	6
InaDl_7	0.56	0.54	36	7
InaDl_8	0.45	0.52	30	6
InaDl_9	0.43	0.52	31	7
LIN7B	0.14	0.31	11	6
MAGI1_3	0.86	0.86	53	27
MAGI1_5	0.75	0.70	14	30
MAGI2_1	0.84	0.86	22	10
MAGI2_4	0.68	0.78	21	15
MAGI2_5	0.68	0.73	20	16
MAGI2_6	0.77	0.79	21	9
MAGI3_1	0.68	0.64	11	12
MAGI3_2	0.78	0.82	11	9
MAGI3_3	0.77	0.82	19	11
MAGI3_4	0.90	0.93	30	12
MUPP1_10	0.69	0.68	39	5
MUPP1_11	0.78	0.83	18	8
MUPP1_12	0.81	0.81	25	8
MUPP1_13	0.86	0.86	42	6
MUPP1_1	0.97	0.98	79	5
MUPP1_2	0.93	0.94	53	5
MUPP1_3	0.94	0.95	53	5
MUPP1_4	0.93	0.95	19	5
MUPP1_5	0.94	0.96	37	5
MUPP1_6	0.8	0.88	12	5
MUPP1_7	0.94	0.96	32	5
MUPP1_8	0.85	0.85	18	6
MUPP1_9	0.93	0.95	37	6
PICK1	0.51	0.56	36	6
PTPN13_PTPL1_PTP1E_1	0.53	0.62	15	5
PTPN13_PTPL1_PTP1E_2	0.63	0.64	35	8
Average	0.71	0.75	-	-

In cases where number of non-binders was much less compared to the number of binders, AUC was calculated by dividing the binders into multiple sets such that, approximately equal number of binder and non-binder were in each set and average AUC values are reported

**Table 2:** Performance of modPDZpep assessed using additional statistical parameters on randomly selected set of equal number of binder and non-binder peptides for 34 domains having ROC-AUC >0.6

Statistical Parameter	Value (Score Cutoff: -2.11)
Sensitivity (%)	68.62
Specificity (%)	70
False Positive Rate (FPR %)	30
Positive Predictive Value (PPV %)	69.58
Accuracy (%)	69.31
F1 Score	0.69

The PDZ domains for which real negative interaction data was not available, artificial negative data [9] obtained by *in silico* approach was used. Additional file 1: Table S1 shows AUC values for ROC- and PR-curves for an additional set of 14 PDZ domains which were benchmarked using artificial negative data. Since this set had more number of artificial negative non-binders compared to true binders for different PDZ domains, here also AUC values for each PDZ domain was calculated by randomly dividing the data into multiple sets, such that each set has equal numbers of binders and non-binders. The final AUC values listed in Additional file 1: Table S1 for each PDZ domain is calculated by averaging over multiple sets. Thus out of the 260 human PDZ domains for which substrates can be predicted by modPDZpep, benchmarking of prediction accuracy could be done for a total of 57 human PDZ domains showing good average ROC-AUC of 0.7. It may be noted that, earlier studies which have used machine learning approach have benchmarked their method on around 27 human PDZ domains [9, 29]. Thus modPDZpep has been benchmarked on highest number of human PDZ domains. All the test data used in the benchmarking are available under benchmark section of modPDZpep web server.

Even though modPDZpep uses residue level scoring scheme, it also provides coordinates of all atom models for PDZ-peptide complexes for detailed analysis of interactions in the binding pocket. These models can be used for re-evaluation of binding energy using atomistically detailed energy functions such as MM-PBSA analysis [30]. modPDZpep uses static crystal structures as templates for modeling the peptides in the binding pocket of PDZ domains and this is based on the assumption that structural variations in the backbone of the PDZ domains and peptide are minimal. We wanted to investigate whether incorporating flexibility by refining the PDZ-peptide using explicit solvent MD simulation can improve the prediction performance. Therefore MD simulations for 5 ns each was performed on all PDZ-peptide complexes for GRIP2_2 PDZ domain and MM-PBSA analysis [30] was performed on MD trajectory to calculate the binding energy values for these complexes. As can be seen in case of GRIP2_2 PDZ domain ranking of peptides based on their MM-PBSA energy values resulted in improvement in ROC-AUC value to 0.63, compared to the ROC-AUC value of 0.47 obtained from pair potential ranking in modPDZpep (Fig. 4c).

Next, we wanted to find out the reason behind differences in prediction performance of our approach on various PDZ domains. First, we analyzed the dependence of substrate prediction performance of modPDZpep on sequence identity of the PDZ domain with the structural templates. We did not find any correlation between the performance of modPDZpep and sequence identity with structural template (data not shown). However, as discussed earlier MM-PBSA results for GRIP2_2 peptide complexes indicate that refinement of the PDZ-peptide complex by inclusion of flexibility for both ligand and receptor leads to better identification of binding pocket residues and this helps in improving the prediction performance. Therefore, it is possible that for certain PDZ domains where conformational flexibility plays a crucial role, modPDZpep (which uses static structures for binding affinity predictions) has low prediction accuracy.

### modPDZpep can perform better on unseen data

modPDZpep was compared with POW (structure based approach) [9] to analyze its predictive power. Predictions can be carried out using modPDZpep for 260 out of 264 human PDZ domains, while POW can predict substrates for only 218 human PDZ domains. Thus modPDZpep has higher coverage of human PDZ domains. Additional file 1: Table S2 lists the 43 PDZ domains used in benchmarking of modPDZpep and also the PDZ domains used in training set by POW. Using real negative data a fair comparison between POW and modPDZpep can only be carried out for 33 PDZ domains for which real negative data has not been used by POW for training (Additional file 1: Table S2). In addition we also included 19 PDZ domains for comparison using artificial negative data, even if artificial negative data for these 19 PDZ domains had been included in the training of POW.

**Fig. 5:**
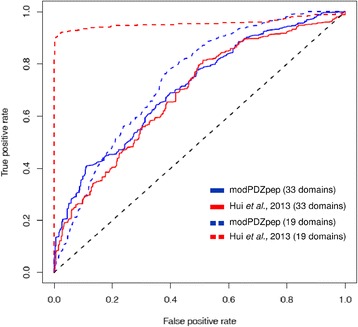
Comparison of performances of modPDZpep and POW (structure based approach) on 33 (combined positive interaction data + real negatives) and 19 PDZ domains (Phage display peptides + artificial negatives). It shows the differential performance of training based methods on the dataset used for training versus data not included in training

Figure 5 shows that when phage display peptides reported by Tonikian et al. [4] are used as binders along with artificial negative data [9] as test set for 19 human PDZ domains (red dashed line), POW had a ROC-AUC of 0.96. However, it must be noted that the same data was used as training set in the POW, while modPDZpep does not use any data for training and hence, achieved ROC-AUC of 0.74. On the contrary, when benchmarking was carried out using binder data for 33 PDZ domains from 4 publications [4, 10, 26, 27] and real negative data as non-binders, modPDZpep outperforms with ROC-AUC of 0.71 as compared to POW which had ROC-AUC of 0.69. The main reason for lower prediction accuracy by POW is that, binding specificity data for these PDZ domains has not been used in training of their method. On the other hand, modPDZpep which is not a training based method performs better on this dataset by using conserved features of protein-peptide complexes. The prediction of interacting partners can be further improved by using all-atom energy functions on the structural models obtained from modPDZpep as we have shown for GRIP2_2 PDZ domain (Fig. 4c). Also incorporation of training into structure based approach of modPDZpep may lead to better performance as shown in a recently published method MSM/D which predicts SH2-peptide interactions utilizing the structural information and machine learning approach [31].

### Development of web interface for modPDZpep

An interactive web based user interface for modPDZpep is available through the URL http://202.54.226.235/modPDZpep.html. It allows the user to model a set of peptides in complex with a PDZ domain and rank them as per their binding energy. Similarly binding affinity of a set of human PDZ domains in complex with a given peptide can also be evaluated. The C-terminal peptide sequence of the putative PDZ interaction partner can be directly entered into the search box or it can be extracted from the UniProt AC and peptide length given as input by the user. The result page of modPDZpep includes a table with BT pair potential score [15] for modeled PDZ-peptide complexes, table of binding pocket residues for highest affinity interaction and downloadable structural models for the PDZ-peptide complexes. It also provides links to SWISS-MODEL modeling report page to get detailed information on template PDZ structures which were used to model the selected PDZ-peptide complex and model quality. modPDZpep also provides option to select and visualize binding pocket and atomic details of any PDZ-peptide structural model using the OpenAstexViewer [32] and JSmol.

Apart from modeling any input peptide in complex with human PDZ domains, as mentioned before modPDZpep also provides graphical user interfaces for analyzing structural details of experimentally identified PDZ-peptide interactions obtained from phage display studies. Currently the known dataset consists of approximately 5400 interactions for 199 human PDZ domains. modPDZpep also provides links to external databases like UNIPROT [25], KEGG GENES [33] and STRING [12] and this helps the user in analyzing additional information about functional interaction network of any human PDZ domain or its putative interaction partner predicted by modPDZpep. An extensive tutorial with detailed explanation on usage of various modules and features of modPDZpep is available on the website.

### PDmapper interface reveals novel PDZ mediated interactions in human protein interaction network

**Fig. 6:**
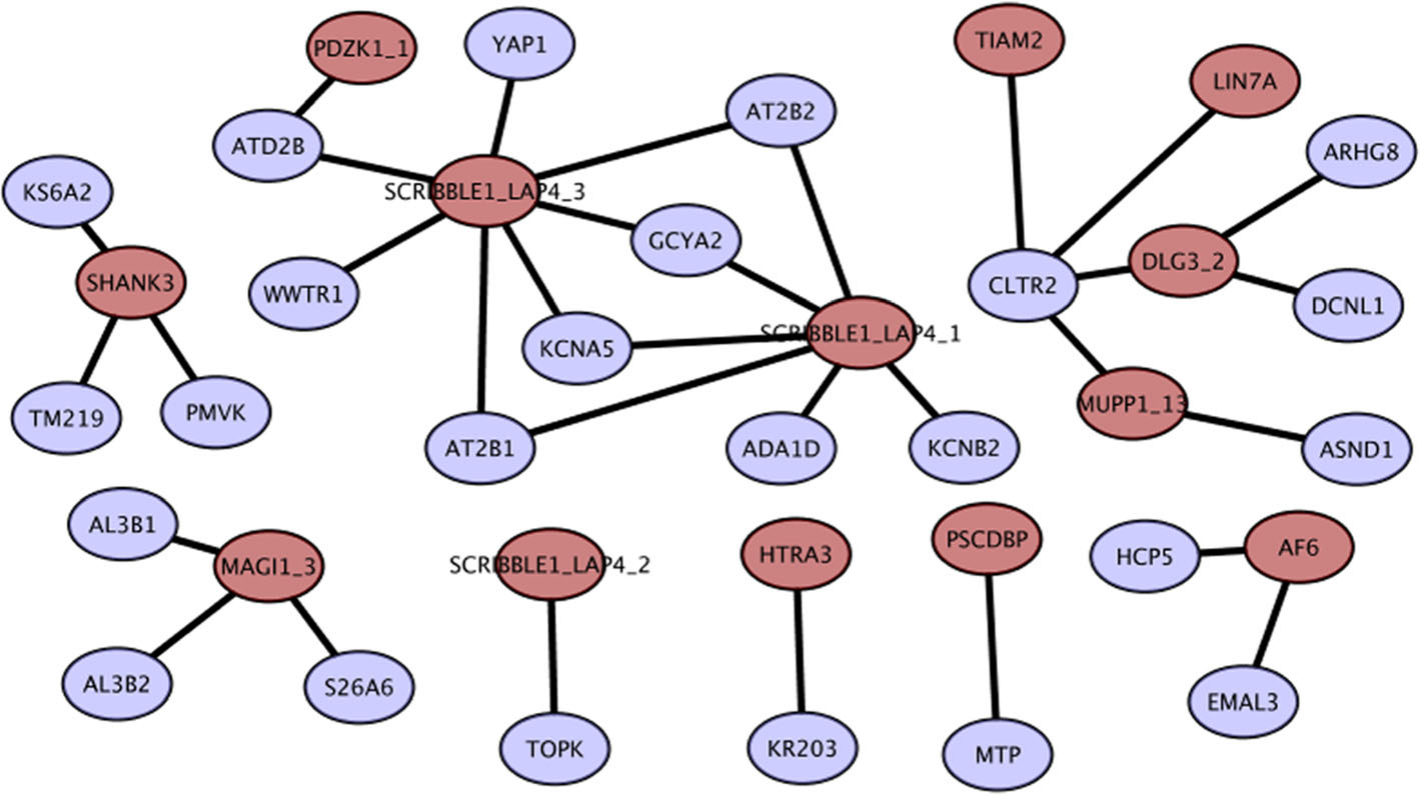
PDZ-protein interaction networks representing novel PDZ mediated interactions identified with PDmapper module of modPDZpep. Red colored nodes depict the PDZ domains and blue colored nodes are the ligand proteins harboring PDZ recognition motifs in the C-terminus based on exact match with PDZ phage display peptides

**Fig. 7:**
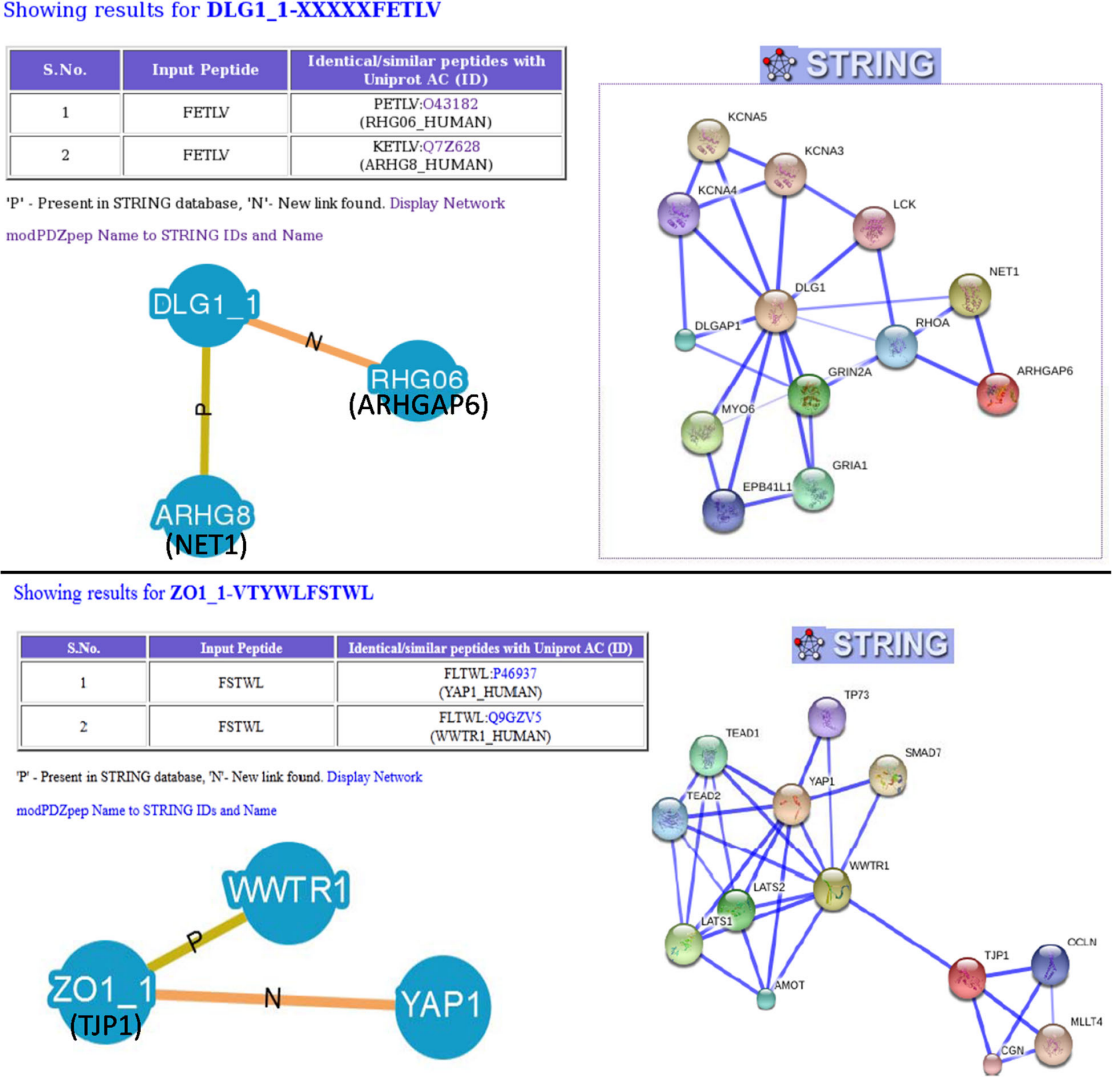
Snapshots depicting utility of modPDZpep in identifying novel direct physical interactions mediated by PDZ domains in Protein-Protein interaction networks available in STRING database. XXXXXFETLV and VTYWLFSTWL were two peptides found to be interacting with first PDZ domains of DLG1 and ZO1 respectively according to phage display data. When the last five residues of these peptides were searched in human C-terminal proteome, both peptides got two hits with one residue mismatch. In case of DLG1_PDZ1, out of the two interaction partners predicted by PDmapper module of modPDZpep, ARHG8 (NET1) is the known interactor present (link indicated by P) in STRING database, but RHG06 (ARHGAP6) which did not have a direct link with DLG1 in STRING is predicted as a new interaction partner of DLG1_PDZ1. Similarly, in case of ZO1 PDZ1 in addition to the known interaction WWTR1, YAP1 is predicted as a new PDZ mediated interaction partner

A large proportion of the experimentally identified interaction partners of human PDZ domains are phage display peptides which may not always correspond to human proteomic peptides. Therefore, PDmapper interface of modPDZpep provides a tool for mapping phage display peptides onto C-terminals of human proteome in terms of exact match or by allowing few mismatches. The PDmapper allows mismatches at any position of the five residue peptide subject to the constraint that C-terminal residue is hydrophobic. When we mapped the human phage display data from Tonikian et al. [4] to C-terminal regions of human proteins, we found exact match for only 45 peptides (Additional file 1: Table S3). Thus these human proteins showing C-terminal matches to PDZ phage display data are bonafide interaction partners of human PDZ domains. Mapping of these PDZ interaction partners onto STRING database revealed 32 new PDZ mediated protein-protein interactions which are not annotated as direct interaction in STRING [12] (Fig. 6). If PDmapper search is carried out allowing one mismatch in the five-residue stretch additional new PDZ mediated interactions can be found in PPI networks. Figure 7 shows examples of direct PDZ mediated interactions between DLG1 and ARHGAP6, and also ZO1 and YAP1 which are not depicted in STRING as directly interacting pairs. This analysis can be done by modPDZpep for any peptide (phage display or predicted binder peptides) using the link to ‘Analyze experimentally known PDZ-peptide interactions’ with variability of one residue and ‘PDmapper’ with variability at any position of 5-residue peptide.

### SNPs can also be visualized on human PDZ domains using modPDZpep

**Fig. 8:**
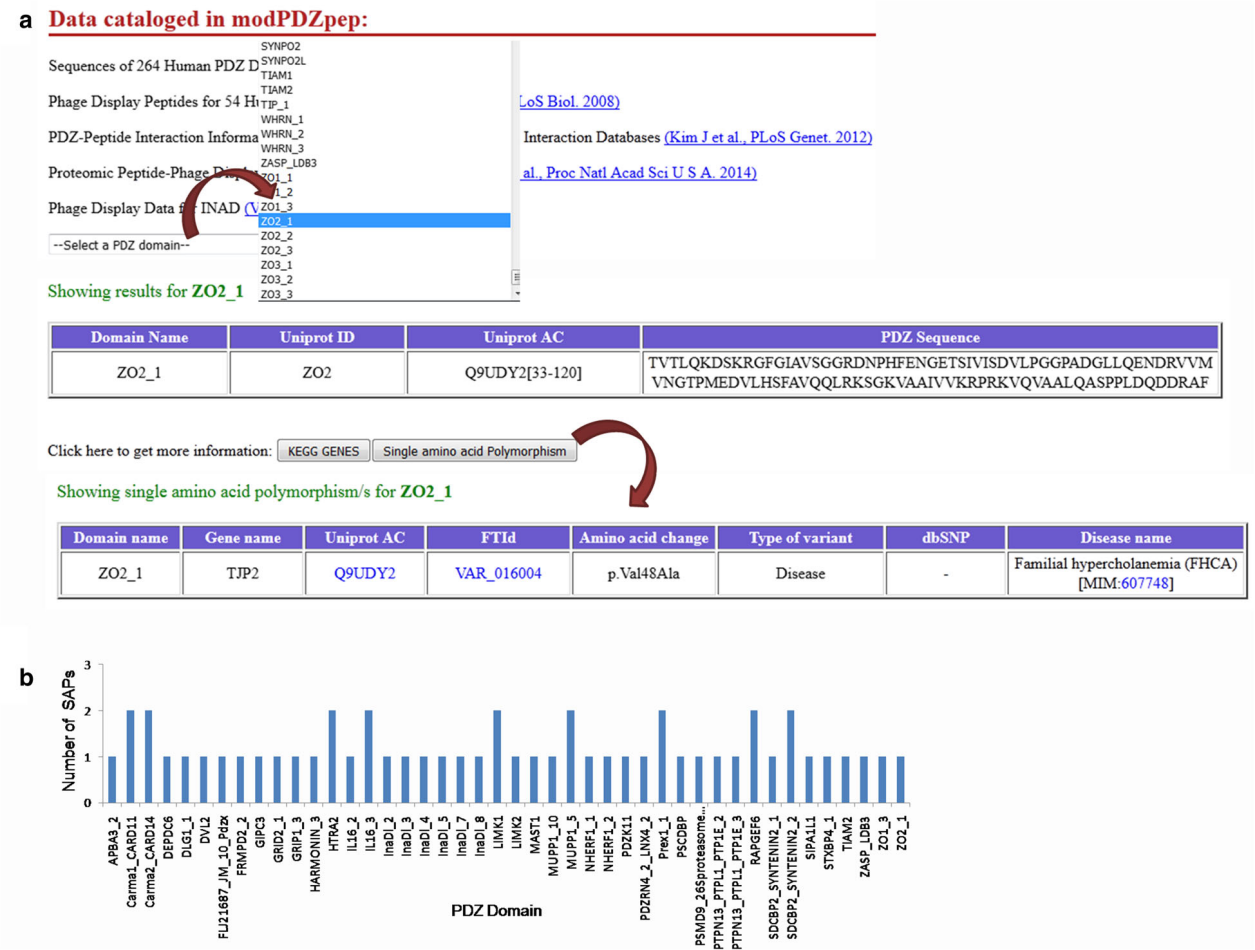
Screenshot depicting SNP analysis of PDZ domains. **a** Upon selecting a PDZ domain, if the non-synonymous SNPs resulting in single amino acid polymorphisms (SAP) are known to occur on the corresponding PDZ domain, modPDZpep shows relevant information i.e. mutation, amino acid position and disease association etc from dbSNP and OMIM. **b** Histogram showing number of single amino acid polymorphisms (SAP) present on various PDZ domains

Single amino acid polymorphisms (SAPs) available in Uniprot were mapped to human PDZ domains (Fig. 8) and we found 53 SAPs to be lying on 44 PDZ domains, out of which 5 are disease-associated SNPs (Additional file 1: Table S4). Even though all these SAPs may not be present in the binding pockets of PDZ domains, they may indirectly affect PDZ-mediated interactions probably by allosteric mechanisms. One such example is of HTRA2-PDZ Gly399Ser SAP which leads to reduced serine protease activity of HTRA2 and neurodegeneration [34].

## Conclusions

modPDZpep provides a platform for structure based analysis of human PDZ domain mediated interaction networks. Given any peptide sequence as input and selecting a human PDZ domain, modPDZpep can provide its modeled structure highlighting the binding interface residues and a score representing the binding affinity between the peptide and the PDZ domain. modPDZPep can predict substrates for 260 out of 264 human PDZ domains, thus it has a better coverage of human PDZ domains compared to other PDZ substrate prediction servers like PDZpepInt and POW. Our detailed benchmarking studies using available experimental data indicate that, performance of modPDZpep is comparable to other machine learning based tools like POW, even if it does not use any data for training. We also demonstrate that performance of structural modeling based approach like modPDZpep can be improved further by using all-atom energy functions and incorporating flexibility to the models of PDZ-peptide complexes. Major advantage of modPDZpep over tools like PDZpepInt and POW is the user friendly graphical user interfaces for analysis of interaction interfaces of the modeled PDZ-peptide complexes. Since all the available phage display data on human PDZ domains are stored in backend databases of modPDZpep, the user can analyze structural basis of recognition specificity for various human PDZ domains. It also provides interfaces for mapping these peptides from phage display studies or other predicted substrate peptides to C-terminal peptides in human proteome and searching the given PDZ-protein pair in PPI databases like databases like STRING. Using this approach, we have identified several novel PDZ mediated interactions in human PPI network. modPDZpep also facilitates analysis of SNPs associated with any human PDZ domains using information from external databases. In summary, modPDZpep will complement the available tools for prediction and analysis of human PDZ networks.

In this work, we have focused on the most prevalent mode of interaction of PDZ domains i.e., C-terminal peptide recognition. While most PDZ domains recognize C-terminus peptides, there are certain PDZ domains which recognize internal peptides [35, 36]. Similarly tandemly occurring multiple PDZ domains on a single polypeptide constitute PDZ supramodules and play important structural and functional role [37]. In case of supramodules interactions between adjacent PDZ domains can affect peptide recognition of constituent PDZ domains because of allosteric regulations. Current version of modPDZpep is not capable of predicting the peptide ligand interaction with PDZ supramodules or binding modes of internal peptides.

## Methods

### Generation of template library for modPDZpep

The sequence of all the PDZ domains in humans were obtained from earlier work by te Velthius et al. [24] for generating the structural templates for all human PDZ-peptide complexes. However, domain boundaries of human PDZ domains were corrected by adding sequence of the N-terminus first β-sheet and 20 residues long extended C-terminal region from Uniprot [25] as these regions were not present in sequences compiled by te Velthius et al. [24]. It has been suggested that the C-terminal extension of PDZ domains often have impact on their interactions [38], therefore the 20 residues stretch at the C-terminus is also included in their sequence which may be useful in detailed structural analysis.

PDZ domain structures were modeled using automated mode of SWISS MODEL [23] if they were not available in PDB. For obtaining the peptide bound models, peptide coordinates were transformed from most similar peptide bound PDZ structure after superimposition of the two structures. It is important to accurately align the two structures for efficient transformation of the coordinates, hence this step is performed with TM-align [39] which can detect fold level similarity. The binding pocket residues of PDZ domain interacting with the peptide were identified from the peptide bound PDZ structure using a distance cutoff of 4.5 Å between any two atoms of a residue pair belonging to peptide and PDZ domain.

### Compilation of high throughput data on substrate specificity of human PDZ domains and compilation of dataset for benchmarking

The high throughput data sets on substrate specificity of human PDZ domains were compiled from phage display studies reported in literature as well as data available in various protein interaction databases [4, 10, 26, 27]. In total, these datasets consist of 5400 interactions for 2898 peptides with 199 human PDZ domains and constitute the positive or binder data. However, after removal of redundant data and exclusion of binder peptides which accommodated any amino acid in any of the terminal five positions, a dataset of 4187 positive interactions for 1907 peptides and 199 PDZ domains was obtained. They have all been stored in backend databases of modPDZpep and are available through ‘Analyze experimentally known PDZ-peptide interaction’ interface in modPDZpep homepage. The majority of the experimental data available for PDZ binding peptides are binary. All the data used for benchmarking in the current study are binary values for binders and non-binders.

The real negative interaction data was taken from the study reported by Luck et al. [40] where they had compiled information from published literature. It provided the 121 non-binder peptides for 79 human PDZ domains defining a total of 573 negative interactions. Both positive and negative data was available for only 66 PDZ domains. However, for benchmarking the prediction accuracy of modPDZpep a dataset of 43 PDZ domains having more than and equal to five binder and non-binder peptides were considered.

### Performance evaluation

We have evaluated prediction accuracy by ROC analysis which involves calculating TP, TN, FP and FN at different values of computed binding energy score cut offs. For this, BT score for every peptide in the dataset was used as cutoff to compute TPR and FPR values. TPR *vs* FPR curve was plotted and the area under the curve (AUC) value was used as performance measure, with higher AUCs indicating better prediction accuracy. AUC value of 1 represents an ideal predictor and 0.5 a random predictor. Apart from ROC curve, Precision-Recall curve was also plotted to assess the performance of modPDZpep. Precision-Recall curve as its name suggests is a curve of precision and recall taken at Y-axis and X-axis respectively.

TPR (True positive rate/Sensitivity/Recall) = TP/(TP + FN)

FPR (False positive rate) = FP/(FP + TN)

Specificity = 1-FPR

Positive predictive value (precision) = TP/(TP + FP)

Accuracy = (TP + TN)/(TP + FP + TN + FN)

F1 score = 2(Precision)(Recall)/(Precision + Recall)

Where TP = True positives, FP = False positives, TN = True negatives and FN = False negatives. The AUC values were computed by PRROC [41] and ROCR [42] package of R.

### Molecular dynamics simulations and MM-PBSA analysis

The GRIP2_2 PDZ-peptide complexes of all 11 binder and non-binder were subjected to molecular dynamics (MD) simulations using ff03 force field of AMBER 12 [43]. Water molecules were used to solvate the complexes in a rectangular box of 8 Å extending from outermost atoms of protein. Then they were minimized by steepest descent algorithm using convergence criteria of 0.001 kcal/mole/Å as RMS gradient of potential energy and equilibrated in two steps. In first step, temperature was increased gradually to 300 K under NVT conditions using langevin temperature equilibration scheme and next pressure was equilibrated for 100 ps. Production dynamics was performed for 5 ns at 2 fs time step under NVT conditions. Particle Mesh Ewald (PME) approach was used to calculate the non-bonded interactions and long-range electrostatic interactions at cutoff of 10 Å. Last 1 ns of simulation was used to extract the snapshots and get the average binding affinity from the trajectories using python script of MM-PBSA analysis [30].

The binding free energy is computed as: ∆G_binding_ = G_complex_ - G_pdz_ - G_peptide_, Where G_complex_ is the free energy for the PDZ-peptide complex, G_pdz_ and G_peptide_ are for the PDZ and peptide respectively.

### Creating human C-terminal proteome

The human C-terminal proteome was generated by downloading the human reference proteome from UniProt [25] and extracting the last five residues of all protein sequences.

### Mapping SAPs on PDZ domains

For SNP analysis, humsavar.txt (release: 2015_08 of 22-Jul-2015) is downloaded from the UniProt [25] which has listed ~71795 single amino acid polymorphisms (SAPs) in human proteins. This document also provides whether a SAP is known to be associated with disease or not. They were mapped to all 264 human PDZ domains.

## Reviewers’ comments

### Reviewer’s report 1: Michael Gromiha, Indian Institute of Technology Madras, India

**Reviewer comments**

**Reviewer summary** In this work, the authors developed a method for predicting the interaction patterns of human PDZ domains using structure based approaches. The structures of complexes have been utilized to predict interaction partners and evaluating binding energy using statistical pair potentials. A web server has been developed for the structure based analysis for application point of view. The manuscript is well written and sufficient details are provided for the analysis and prediction.

**Reviewer recommendations to authors**

It could be strengthened by incorporating the following suggestions.

1. The residue pair potentials have been used to compute the binding energy. It will be helpful to provide the details about BT potentials, obtaining the binding energy from BT pair potentials and the contributions in DGbinding.

Author’s response: *BT (Betancourt-Thirumalai) pair potential is a knowledge based scoring function derived from observed contact frequencies between various amino acid residues and expressed as 20x20 contact potential matrix corresponding to all possible amino acid pairs. The binding energy for each PDZ-peptide complex is scored as sum of the interactions between all residue pairs between the peptide and PDZ domain which are at a distance less than 4.5 Å. The binding energy score computed using residue based pair potential is assumed to correlate with binding free energy in a low resolution model, thus it is a suitable scoring function for discriminating potential binders from non-binder peptides. However, our earlier studies have indicated that high resolution models with atomistic scoring functions are required for quantitative correlation with experimental binding free energie*s.

2. It is not clear whether the experimental data are binary or real values. If the real values are known the performance may assessed with correlation coefficient and mean absolute error. It seems the binding energy data are in numerical values and it will be better to explain the procedure to convert into binaries (any cutoff values) to compute TP, TN, FP and FN.

Author’s response: *The majority of the experimental data available for PDZ binding peptides are binary. All the data used for benchmarking in the current study are binary values for binders and non-binders. Real values of binding affinities are available only for few PDZ domains and in an earlier study from our group we have used that data to evaluate correlation coefficient between predicted and experimental binding energies*.

*We have evaluated prediction accuracy by ROC analysis which involves calculating TP, TN, FP and FN at different values of computed binding energy score cut offs. For this, BT score for every peptide in the dataset was used as cutoff to compute TPR (=TP/TP + FN) and FPR (=FP/FP + TN) values. TPRvs FPR curve was plotted and the area under the curve (AUC) value was used as performance measure, with higher AUCs indicating better prediction accuracy. AUC value of 1 represents an ideal predictor and 0.5 a random predictor.*

3. Several decimal points are not necessary in Table 1.

Author’s response: *We have modified Table**1**and rounded off values to two digits after decimal*.

4. Experimental data should be given at the website.

Author’s response: *We have provided the experimental data compiled for this study under the link ‘Analyze experimentally known PDZ-peptide interactions’ in modPDZpep web server*.

5. The performance may be compared with other methods in the literature although they use experimental data for training.

Author’s response: *The performance of modPDZpep has been compared with the other structure based method POW* [9] *which uses experimental data for training. These results are shown in Fig.*5*. modPDZpep is found to have better performance on unseen data, while on datasets which have been used for training of POW, the performance of POW was higher compared to modPDZpep*.

6. Molecular dynamics simulations have also been performed to obtain the binding energy. It is not clear about the applications of MD in prediction.

Author’s response: *modPDZpep uses static crystal structures as templates for modeling the peptides in the binding pocket of PDZ domains and this is based on the assumption that structural variations in the backbone of the PDZ domains and peptide are minimal. We wanted to investigate whether incorporating flexibility by refining the PDZ-peptide using explicit solvent MD simulation can improve the prediction performance. Therefore MD simulations for 5 ns each was performed on all PDZ-peptide complexes for GRIP2_2 PDZ domain and MM-PBSA analysis was performed on MD trajectory to calculate the binding energy values for these complexes. Ranking of peptides based on their MM-PBSA energy values resulted in improvement in ROC-AUC value for GRIP2_2 PDZ domain compared to the ROC-AUC values obtained from pair potential ranking in modPDZpep*.

### Reviewer’s report 2: Zoltán Gáspári, Pazmany University, Budapest

**Reviewer comments**

**Reviewer summary** The manuscript describes a method to estimate the binding affinities of peptides to human PDZ domains. The method is based on structural modeling of the complexes. The approach is interesting and combines previous results in a meaningful and usable manner. The corresponding web service is well designed and offers useful functionality. Overall I think that the workflow has its merits and is of importance in its field.

**Reviewer recommendations to authors**

I have remarks about the presentation and discussion of the results.

- The study implies that backbone structural variations both in the PDZ domain and the bound peptide are negligible. It would be advisable to provide a justification of this premise.

Author’s response: *Our approach is based on the assumption that backbone structural variations both in the PDZ domains and the bound peptides are minimal. Analysis of crystal structures of PDZ domains and PDZ-peptide complexes in earlier studies from our group as well as others* [11, 14] *have revealed that, backbone RMSDs of PDZ domains show good correlation with sequence similarities. It has also been shown that backbones of bound peptides superpose well when corresponding PDZ domains are superimposed. Therefore, in coarse grained modeling of PDZ peptide complexes for quick screening of binding partners assumption about minimal structural variations is justified. However, this does not imply that conformational flexibility of PDZ-peptide complexes have no role in peptide recognition. As explained in answer to the next question, for certain PDZ domains where performance of modPDZpep is not good, conformational flexibility might be playing a significant role*.

- Can the authors offer any (structure-based) explanation for the differences in performance on different PDZ domains?

Author’s response: *Our structure based approach relies on accurate modeling of the structure of the PDZ domains and identification of the correct binding pocket residues. We have analyzed the dependence of substrate prediction performance of modPDZpep on sequence identity of the PDZ domain with the structural templates. We did not find any correlation between the performance of modPDZpep and sequence identity with structural template (data not shown). However, when we performed the MD simulation and MM-PBSA analysis on one of the PDZ domains i.e., GRIP2_2 peptide complexes, we noted improvement in ROC-AUC. This implies that refinement of the PDZ-peptide complex by inclusion of flexibility for both ligand and receptor leads to better identification of binding pocket residues and this helps in improving the prediction performance. Therefore, it is possible that for certain PDZ domains where conformational flexibility plays a crucial role, modPDZpep (which uses static structures for binding affinity predictions) has low prediction accuracy*.

- Please comment on the relation of the approach and known variations of PDZ:peptide binding modes, with emphasis on PDZ-PDZ supramodules (e.g. Feng & Zhang, 2009, Nat Rev Neurosci 10:87).

Author’s response: *There are other known non-canonical modes of recognition for PDZ domains like interaction with internal peptides. While most PDZ domains recognize C-terminus peptides, there are certain PDZ domains which recognize internal peptides* [35, 36]*. Similarly, tandemly occurring multiple PDZ domains on a single polypeptide constitute PDZ supramodules and play important structural and functional role. In case of supramodules interactions between adjacent PDZ domains can affect peptide recognition of constituent PDZ domains because of allosteric regulations* [37]*. In this work, we have focused on the most prevalent mode of interaction of PDZ domains i.e., C-terminal peptide recognition. We generated the PDZ domain structural models alone without considering the adjacent domains, hence modPDZpep is not capable of predicting the peptide ligand interaction with PDZ-PDZ or any other supramodules. Similarly current version of modPDZpep cannot model binding modes of internal peptides. However, we have structures of some PDZ domains e.g. ZO2_2, Periaxin etc. as homo-dimers in the template library which are known to dimerize through domain-swapping*.

- Please provide a more robust justification for using the BT pair potential instead of referring to its application in your previous work also.

Author’s response: *Residue based statistical pair potentials are less compute intensive and allow the fast screening of potential peptide substrates of PDZ domains and are thus suitable for genome scale analysis interaction network. A number of different residue based statistical pair potentials are available in the literature and they have all been derived from observed contact frequency of different pairs of amino acids in a non-redundant set of crystal structures in PDB. Basic assumption in derivation of all these pair potentials is that the observed contact frequency of an amino acid pair when normalized with respect to the expected frequency in a suitable reference state would present interaction energy between the residue pair. The major difference in various pair potentials is in the choice of reference state. Miyazawa-Jernigan (MJ) potential was derived using solvent as reference state while Betancourt-Thirumalai (BT) pair potential used a solvent like molecule Thr as reference state. Therefore, it has been suggested that while MJ matrix gives more weightage to hydrophobic interactions, BT matrix can also represent hydrophilic interactions more accurately. In our earlier study BT matrix was found superior to MJ matrix in identification of interaction partners of MHCs, kinases and mouse PDZ domains*.

**Minor issues**

- Please refrain from using the phrase “most homologous”, use “most similar” instead. Please comment on whether here overall similarity or local similarity (in and near the binding site) can yield more reliable results and please state explicitly how you used and measured the similarity in this respect.

Author’s response: *The phrase “most homologous” has been replaced by “most similar” throughout the manuscript. We thank the reviewer for pointing this out*.

*In this work we have calculated sequence similarity over the entire length of the PDZ domain. For a given human PDZ domain, the crystal structure having highest sequence similarity has been used a template for structural modeling. Our work is based on the assumption that high degree of overall sequence similarity also implies high degree of local similarity in the binding pocket region. Based on this assumption in case of PDZ crystal structures lacking bound peptides, the peptide from the structures having highest overall similarity have been transformed after aligning the PDZ domains using TM-align software.*

*However, we agree with the reviewer that binding pocket similarity might be more useful and correlation between overall similarity and binding pocket similarity need to be analyzed for PDZ domains in details.*

- Please explain in detail how exactly the 98 % estimate of the human PDZome and its interactions potentially covered by the approach was obtained.

Author’s response: *The human PDZ sequences retrieved from te Velthius et al.* [24] *were mapped to Uniprot accession numbers and we found Uniprot accession numbers for 264 human PDZ domains in total. Out of these 264 human PDZ domains, structural models could be built for 260 PDZ domains and modPDZpep server can predict potential interaction partners for these 260 human PDZ domains using structure based approach. Since modPDZpep can predict interaction partners for 260 out of 264 human PDZ domains, we have mentioned that it can predict interaction partners for ~98 % of human PDZome*.

-In Additional file 1: Table S1, please give the minimum and maximum ROC values and the standard deviation besides the averaged value.

Author’s response: *The values for maximum and minimum AUC as well as corresponding standard deviations have been added in Additional file**1**: Table S1*.

- I suggest to combine the Supplementary tables into a single Excel file with multiple tabs.

Author’s response: *All Supplementary tables have been combined into EXCEL single file with multiple tabs*.

## Additional file

Additional file 1: All Supplementary tables are merged into single Excel file. **Table S1.** ROC- and PR-AUC values for 14 Human PDZ domains with artificial negative interaction data. Approximately equal number of binder and nonbinder were considered in small sets and average AUC is calculated for each domain. For SCRIBBLE1_LAP4_3, PSCDBP and NHERF2_2, equal number of non-binder peptides were randomly selected and AUC is calculated. This is repeated twice to get average AUC. **Table S2.** List of 43 PDZ domains included in modPDzpep testset. PDZ domains which cannot be predicted by POW are marked as ‘N’, while those used in the training are marked ‘T’. The list of test set peptides for these 43 PDZ domains is available under **Benchmark** link of modPDZpep server. **Table S3.** List of phage display peptides which showed exact match in human C-terminal proteome **Table S4.** Amino acid variants present on human PDZ domains. (XLS 55 kb)

## Abbreviations

AUC: Area under curve
BT pair potential: Betancourt-Thirumalai pair potential
PPI: Protein-protein interaction
PR: Precision-recall
PRM: Peptide recognition module
ROC: Receiver operating characteristic
SAP: Single amino acid polymorphism.
